# The Evolution of External Healthcare Regulation in England; From Performance Oversight to Supporting Improvement: Comment on "The Special Measures for Quality and Challenged Provider Regimes in the English NHS: A Rapid Evaluation of a National Improvement Initiative for Failing Healthcare Organisations"

**DOI:** 10.34172/ijhpm.2023.7809

**Published:** 2023-02-27

**Authors:** Mirza Lalani, Helen Hogan

**Affiliations:** Department of Health Services Research and Policy, London School of Hygiene and Tropical Medicine, London, UK.

**Keywords:** Quality Improvement, Healthcare Regulation, Whole System Approaches, England

## Abstract

The Special Measures and Challenged Provider (SMCP) Regime introduced for struggling healthcare organisations in England represents a subtle shift to the scope of external regulation from performance oversight to include supporting internal service improvement. External regulation alone has a had a mixed impact on the quality of care and Vindrola-Padros and colleagues’ study highlights that externally driven improvement initiatives may also struggle to succeed in turning around performance. Principally, this is due to a failure in acknowledgment that poor performance results from a myriad of external and internal factors which coalesce to impede organisational performance. A struggling organisation may be indicative of wider issues in the local health and care system. Whole systems approaches to improvement with collaboration across providers and the effective use of data may support struggling organisations but their role maybe tempered with the increased centralisation of the delivery of improvement regimes such as SMCP.

 The publication of Vindrola-Padros and colleagues’ paper^1^ on Special Measures and Challenged Provider (SMCP) regimes in the English National Health Service (NHS) provides a timely reminder of the challenges in maintaining and improving the quality of care. The work provides novel insights into the role of externally driven improvement initiatives in supporting struggling NHS organisations and their association with both positive and negative consequences. It is an extensive study spanning several types of NHS organisations and involves a range of participants providing a detailed and nuanced picture of SMCP regimes. The adoption of rapid qualitative methods in this type of work is novel in the context of health services research in the United Kingdom. The rapid qualitative approach provides a means of undertaking research and evaluation of new policy initiatives at pace, identifying factors affecting implementation and early insights on impact. This provides policy makers with the opportunity to review the continuing relevance of the initiatives and adapt to the continually changing care landscape.

## Improvement Regimes

 National improvement/performance management programmes in England emerged in the early 2000s in response to prominent quality of care failures in NHS organisations. External quality monitoring was implemented by a succession of regulatory bodies from the Centre for Healthcare Improvement followed by the Healthcare Commission and then the Care Quality Commission (CQC). These bodies were charged with identifying the few “bad apples” across the NHS. They employed a range of approaches, influenced by the prevailing political context of the time, to bring quality outliers into line. Alongside inspections, the Centre for Healthcare Improvement developed star-ratings according to the performance of NHS hospitals against national targets. Those that were zero-rated or failing on inspection were publicly named (and shamed). Better performing organisations were rewarded with ‘earned autonomy.’^2^ This regime was superseded by the Annual Health Check, which adopted a more comprehensive approach to assessment and inspection, incorporating core and developmental standards in areas such as patient focus and the healthcare environment.^3^ The formation of the CQC signalled a more relational approach, gradually shifting the balance of assessment from intermittent inspection to regular contact, illuminating more clearly local organisational context and culture.

 External regulation was not limited to organisations. Following much debate, propagated by the Shipman Inquiry, Medical Revalidation was introduced in 2012 to address longstanding concerns about the accountability of doctors and the quality of medical care. It involved assessing a doctor’s performance through collection, reporting and reflection on relevant information produced for annual appraisal.^4^ Looking back, external regulation has had a mixed impact on the quality of care delivery. At the professional level, medical revalidation may have stimulated improvements in clinical governance and clinical practice for doctors whose practice had raised concerns, but its value has been less clear in further improving the practice of well performing doctors.^4^ At the organisation level, targeting struggling organisations who are reliant on internal clinical governance processes to promote improvement, has exposed flaws in external regulation exemplified by high profile patient safety failures in several NHS organisations. Star ratings were thought to have improved performance eg, reducing accident and emergency waiting times but were criticised for aggregating diverse performance indicators into a single performance score obscuring where intervention may be needed, a lack of transparency of the scoring system and a detrimental effect on staff morale of a zero rating.^5^ The Annual Health Check was considered to have improved standards, with evidence of inspections triggering change, yet, its self-assurance component resulted in only 20% of hospitals being inspected annually (mainly based on estimates of risk). Despite CQC’s more frequent and wide-ranging inspection regime, it is subject to variation in the reliability of the inspector’s judgement as well as having a limited impact on raising performance standards and improving the quality of care.^6^ The SMCP regime is designed to be supportive and encourage relationship building between the CQC and struggling Trusts and represents a departure from the traditional remit of an external regulator, extending beyond performance oversight and regulation, to include direct intervention to stimulate improvement.

## Why Do National Improvement Programmes Struggle to Deliver Improvement in Quality?

 Of the 40 NHS hospitals that have been under the SMPC regime between 2013-2018, only six have been rated as ‘good’ subsequently, with the same number re-entering the regime.^7^ Vindrola-Padros et al findings highlight a range of reasons why externally driven improvement initiatives often do not succeed in achieving sustained performance improvement. Firstly, the premise that failures in the NHS are down to a relatively small number of outlying NHS organisations that can be identified by a combination of inspection and performance data and then remedied, is flawed. NHS hospitals are complex systems made up of multiple components whose quality may range from excellent to poor. Furthermore, poor performance results from a myriad of external and internal factors such as absent or dysfunctional technology, management bureaucracy, staffing, financial constraints or other issues unique to an organisation which coalesce to impede organisational performance.^8^ Given such complexity, the reliability of regulators’ diagnosis of poor performance is questionable. Targeting support in this way is a blunt instrument, potentially unfairly stigmatising some NHS organisations whilst others, with perhaps even poorer performance in certain areas, remaining under the radar. Moreover, additional support for improvement may end up sub-optimally distributed across the NHS.

 If there are issues with the detection of quality problems, then improvement initiatives are more likely to be poorly targeted, limiting their capacity for success. Vindrola-Padros et al found such initiatives may have both positive and negative outcomes. As a consequence, service improvement in complex organisations following interventions cannot be assumed.^9^ Improvement initiatives are not without costs – the addition of extra tasks for staff without clear rationale and obvious benefit, may further contribute to staff burden at a time in which burnout is prevalent across the workforce.^10^ The need to provide enhanced performance data whilst within the programme, uses up further resources especially when the organisation has limited power and capacity to change the drivers of key performance indicators. Entering an improvement regime has wide-ranging impacts for NHS organisations. It is not surprising then that Vindrola-Padros et alfound that staff viewed the SMPC approach as punitive, especially in its preliminary phase. The unintended consequences of reputational damage can lower staff and patient morale. Future risks to quality and safety are heightened if such damage leads to lower ability to attract and retain staff.

 Interventions such as SMCP are short term fixes aimed at halting the decline in care quality and beginning the gradual process of reversal in individual organisations. Vaughn et al posit several of the issues highlighted in this study that limit improvement potential in struggling organisations, including poor culture, inadequate infrastructure, lack of a cohesive purpose, system shocks and impaired relationships with other organisations.^11^ Such issues are often longstanding and worsened by contributory factors that lie outside the organisations. Quality improvement is a long-term commitment whose benefits may only be realised if changes are adopted and sustained in the organisation’s governance and leadership, capacity and culture. The latter requires a mindset shift to recognise the value of improvement work at all levels and the creation of a just culture.^12^ Moreover, identification of external factors driving internal problems and taking a systems-based perspective to their solution is also key. We know relatively little about why some SMCP hospitals continue to struggle in a perpetual cycle of inadequate performance whilst others make considerable improvement. A qualitative unpacking of the journey of these organisations post SMCP would provide insights about the utility of improvement initiatives in the longer term and ensure additional support is available at the earliest sign of performance regression.

 Vinrdola-Padros et al findings draw attention to the importance of senior leadership in improvement, particularly in securing resources and engaging staff. The SMCP regime is a reminder that allocating responsibility for improvement to a single individual and short-term leadership are not a panacea. Sustained change where quality improvement is embedded into routine practice and seen as everyone’s responsibility requires consistent, collaborative and diverse leadership at all levels of an organisation.

## Looking to the Future and the Role of the System

 A struggling organisation maybe a symptom of wider local system malaise. Acute providers may suffer an adverse impact on performance when primary care is overburdened resulting in an increase in emergency and urgent care attendance compounded by delayed discharge of convalescing patients to the community because of backlogs in social care. There is scope for a whole-systems approach to supporting struggling organisations, thereby addressing system-wide performance problems. Integrated Care Systems (ICS) in England have heralded opportunities for organisations to engage in collaborative working across care boundaries, sharing resources, instituting mechanisms that promote peer learning to share good practice and developing collective accountability. Whole systems approaches that provide comprehensive and transparent data from across a range of sources (primary, secondary, community and social care) supplemented with qualitative insights have the potential to promote a more proactive approach to identifying unwarranted variation earlier in its gestation. Ultimately, the availability of better quality data enabling earlier targeted responses to emergent problems may pave the way for simplified approaches to assurance and regulation and reduce the need of multiple external regulatory bodies.

 Central bodies are adapting to this recent restructuring. The CQC have proposed a single assessment framework assigning performance ratings to systems using a broader array of quality of care outcomes across safety, experience, equity and access while also examining progress on integration such as partnership working and public/patient involvement.^13^ NHS England (NHSE) has strengthened its regional teams to facilitate continuity in monitoring performance so that issues can be identified issues at an earlier stage. This should allow better tailoring of the scale and scope of support required by organisations and systems.^14^

 Current NHSE systems oversight includes a Recovery Support Programme Framework which provides NHSE with discretion to directly intervene to performance manage providers with quality or financial issues.^15^ The Recovery Support Programme is similar to SMCP including a short-term improvement director, regular progress and challenge meetings hosted by NHSE regional teams, an appointed Trust Board advisor and enhanced reporting and controls.^14^ Crucially, the initiative now requires system partners to provide support which should enable provider issues to be tackled with better awareness of local context and constraints and strengthen relationships between NHSE/CQC and systems/organisations promoting collaborative working across a system. However, there is a danger for NHSE in direct performance management of individual providers, rather than providing support to systems to tackle these issues themselves, as any improvements may be limited in scale and sustainability.

 England is not alone in attempting a transition from accreditation, external regulation and oversight to an outcomes focussed improvement approach. In 2015, Denmark launched a National Quality Programme centred on driving improvement through the continuous and transparent use of data accessible for all stakeholders. Evidence of impact of the programme is promising, with performance indicators showing improvement in clinical outcomes in mental health, cancer and cardiovascular disease and in service outcomes with a fall in hospital length of stay.^16^ Crucially, responsibility and accountability has been devolved to local organisations. This draws parallel with the long-term tacit intentions for ICS in England that advocate for the availability of more dynamic data at the local level, decentralising responsibility thereby reducing the need for external oversight. Such a transition will require a significant cultural shift in a healthcare system accustomed to centralisation and diktat.

## Conclusion

 The SMCP regime is emblematic of a changing approach to external regulation in healthcare in England centred on collaboration with organisations, professionals and patients with the goal of facilitating improvement, particularly in struggling organisations. Improvement takes time, requires resources and commitment, leadership with the necessary expertise and staff engagement. The sheer complexity of healthcare organisations means substantive change is not guaranteed, especially in the short term.

 A whole systems approach to improvement with collaboration and the effective use of data should enable earlier detection and more targeted support to individual organisations delivering poor quality care. The establishment of ICS presents an opportunity to change the nature of the relationships between regulators, systems and providers from a top-down assurance-based approach to one of collaboratively supporting improvement. There are potential risks of blurred lines of accountability when an organisation fails and ongoing research and evaluation of the new oversight arrangements particularly the role of the system and NHSE in performance managing failing organisations, will be required.

## Ethical issues

 Not applicable.

## Competing interests

 Authors declare that they have no competing interests.

## Authors’ contributions

 ML and HH jointly conceived and drafted the commentary before agreeing on a final version.
